# Correction: Ventilation during COVID-19 in a school for students with intellectual and developmental disabilities (IDD)

**DOI:** 10.1371/journal.pone.0313792

**Published:** 2024-11-08

**Authors:** Martin S. Zand, Samantha Spallina, Alexis Ross, Karen Zandi, Anne Pawlowski, Christopher L. Seplaki, Jonathan Herington, Anthony M. Corbett, Kimberly Kaukeinen, Jeanne Holden-Wiltse, Edward G. Freedman, Lisette Alcantara, Dongmei Li, Andrew Cameron, Nicole Beaumont, Ann Dozier, Stephen Dewhurst, John J. Foxe

An error was identified in S1 File of this article [1], resulting in a portion of the Room_Type column and the entire Mean_Occupancy column containing entries for the room record index numbers, instead of the correct data. This occurred due to a formatting error. The updated S1 File is provided here. The authors confirm that this error does not affect the reported results or conclusions.

In addition, concerns were raised after [1] was published that the linear regression in Fig 8B is not appropriate for the analysis of the correlation between hours of classroom CO_2_ levels and incidence of SARS-CoV-2 infection. The linear regression analysis was removed and Fig 8 has been updated here. Additionally, the ninth sentence of the Incidence of SARS-CoV-2-positive PCR tests section of the Results is corrected to:

“A weak correlation was found between normalized positive PCR tests per classroom and hours of classroom CO_2_ levels ≥1,000 ppm (Spearman rank correlation; p = 0.01937, *ρ* = 0.2864), but not estimated classroom ACH (p = 0.3912, *ρ* = 0.0876).”

A member of the *PLOS ONE* Editorial Board reviewed the updated figure and underlying data for the experiments and stated that the conclusions in [1] are still supported.

To expand on the limitations of [1], the authors also replace the last sentence of the fourth paragraph of the Discussion with the following:

“It is also important to note some study limitations. We did not measure individual exposure over time to CO_2_ concentrations (e.g. fitting each subject with a CO_2_ sensor), but rather room concentrations. Individuals generally move between classrooms, therapy rooms, the outdoors, and other school areas, although less so during COVID, and CO_2_ exposure may vary from estimates by room dwell times. In addition, this was a real-world study and data collected on individuals was limited. This limited more complex analyses that could adjust for potential predictors or confounders of the number of positive PCR tests per room, especially those related to individual occupants (e.g. immunosuppressed status, SARS-CoV-2 exposure histories outside of school, SARS-CoV-2 immunity, masking). More comprehensive epidemiological studies, which include detailed contact tracing and prospective subject monitoring, could be undertaken in the future to address this limitation.”

During their review of the article and its concerns, the Editorial Board member also advised that the linear regression was not appropriate due to limited information on other predictors and confounders of SARS-CoV-2 infection, which is reflected in the limitations statement included by the authors. The significance of the relationship between the number of positive PCR tests of SARS-CoV-2 infection per room occupancy and CO_2_ >1000 ppm was determined by Spearman’s rank correlation, which was weak but statistically significant. They also confirmed that these corrections support the results and conclusions reported in the article.

The authors apologize for the errors in the published article.

**Fig 8:**
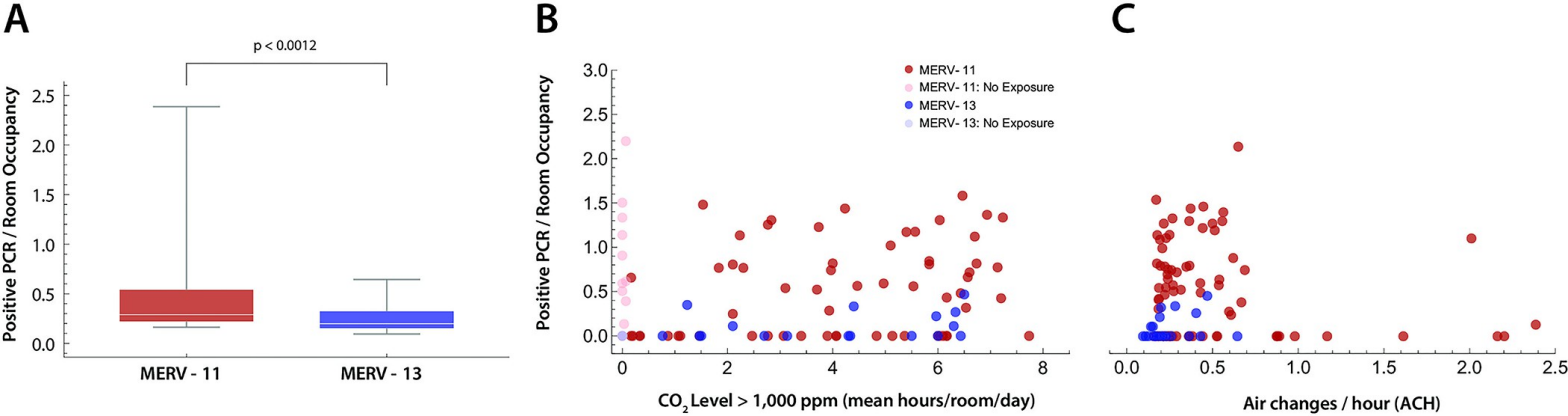
Positive PCR tests per room. (A) Difference in PCR tests per room, normalized by room occupancy, between rooms with HVAC systems using MERV-11 (red) versus MERV-13 filters. Statistical comparison with the Mann-Whitney U-test (p<0.0012). (B) The time in each room where the ambient CO_2_ ≥ 1,000 ppm was plotted against positive PCR tests in that room normalized by mean room occupancy. Rooms with CO_2_ ≥ 1,000 ppm for less than 0.1 hour were excluded (lighter markers). MERV filter status for the building rooms is shown (red, MERV-11; blue, MERV-13). Spearman rank correlation (*ρ* = 0.2864) showed a weak correlation. (C) ACH versus positive SARS-CoV-2 PCR tests per room normalized to room occupancy. No correlation was found (*ρ* = 0.0876).

## Supporting information

S1 File Room Data. A CSV file containing data with room characteristics, 362 including building, square footage and volume, occupancy, CO_2_ concentration times, 363 SARS-CoV-2 PCR positive subjects per room.
(CSV)
