# Supplementary material for: Ventilation during COVID-19 in a school for students with intellectual and developmental disabilities (IDD)
Source: PLoS One. 2024 Apr 3;19(4):e0291840. doi: 10.1371/journal.pone.0291840 (PMC10990219; doi:10.1371/journal.pone.0291840)
Supplement: Attachment — Submitted filename: PLoS Rev Full reorg.pdf [file pone.0291840.s004.pdf]

Response to Reviewers: PONE-D-23-26827: Ventilation during COVID-19 in a school for students with intellectual and developmental disabilities (IDD)

N.B. Please note that all line references [e.g. L25-31] refer to lines in the marked up PDF, not the clean copy.

Reviewer #1: *The research article indicates the study examined the correlation of classroom ventilation (air exchanges per hour (ACH)) and exposure to CO2 ≥1,000 ppm with the incidence of SARS-CoV-2 over a 20-month period in a specialized school for students with intellectual and developmental disabilities (IDD). Different ways of mitigating this issue is well explained. Statistical analysis and the figures are depicted appropriately. Further the experimentation as per the research is accomplished for the set purpose. the conclusion drawn is structured and provides some evidential inferences. Henceforth the research article is strongly accepted for publishing.*

We thank reviewer #1 for their comments, which do not recommend any changes.

N.B. Reviewer #2 included over 100 separate bullet points, a number of which were comments and not requests for revisions. For clarity, we have consolidated all the points, and only included those below that requested changes in the manuscript.

Reviewer #2: *This article (Ventilation during COVID-19 in a school for students with intellectual and developmental disabilities (IDD)) investigates SARS-CoV-2 transmission in an IDD school and its relationship with ventilation and CO2 levels. Findings reveal that less than 20% of staff and students had PCR-confirmed SARS-CoV-2 infections, consistent with previous school studies. CO2 monitoring is highlighted as a potential tool to assess ventilation adequacy, with elevated CO2 levels impacting cognitive performance and health. The study emphasizes the need for improved ventilation and air filtration in schools and discusses the associated trade-offs, particularly for IDD-specialized schools. Public funding and policy discussions are suggested to address these challenges and enhance safety in educational settings. However, the authors could address the following suggestions for improving the quality of the article:*

<u>Literature Review:</u> *Could include more recent research on SARSCoV2 transmission in schools.*

The literature on SARS-CoV-2 in schools is, as noted, extremely sparse. We have updated the references to include recent references [L12-14 and L30-34].

<u>Specific Aims and Research Gap:</u> *Could benefit from explicitly stating the research aims. Needs a clearer statement of the research gap in the existing literature regarding ventilation and SARSCoV2 in IDD specialized schools.*

We have revised the introduction to explicitly state the research aims [L12-14]. We have also added a statement that, to our knowledge, there is no literature regarding ventilation and SARS-CoV-2 in IDD Schools [L32-34]

<u>NIH RADxUP Program:</u> Might benefit from a brief description of the project's objectives within this context.
We have revised the manuscript to frame the project's objectives. [L76-81]

<u>Room and Building Data:</u> Information about HVAC systems and their significance would be helpful.
Significance of HVAC systems, in general, was provided in the original manuscript in the introduction [L16-30]. Information linked to each HVAC system was provided in Supplemental File S1, linked to each room [L99]. We have revised the manuscript to provide some framing information about HVAC systems and their significance in ventilation [L207-208; L211-212]

<u>SARSCoV2 Case Data:</u> Defined the timeframe for data collection but could include the rationale.
Respectfully, we did not have a choice regarding timeframe. SARSCoV2 case data collection began when NY State mandated reporting for schools and the , and we selected a 1 year period after this. Given the nature of the pandemic, we would not have been able to accurately "select" a timeframe prospectively for data collection.

CO2 Measurements: *More information about the rationale for the specific measurement date might be beneficial.* We have revised the manuscript to include an explanation for the selected measurement date range [L109-110].

Room Characteristics: *Explanation of the significance of HVAC systems and MERV filters might enhance understanding.* As noted above, we have added explanations of HVAC systems and MERV filters [L165-169].

CO2 Level Profiles: *Could benefit from explaining the significance of these patterns.* We are unclear which patterns the reviewer is referring to: the time-varying patterns of the measurements, the pattern of distribution within the buildings. The variation in patterns is already discussed, with notes regarding the significance (drowsiness, adherence to recommended standards). Thus, we have not modified this section.

Room CO2 Concentrations: *Explanation of the chosen CO2 ranges and their relevance is suggested.* Respectfully, the manuscript provides references to the CO2 ranges [L189; L191; L194]. The CO2 ranges are, as referenced, those recommended by national standards in the US by ASRHE and OSHA, as noted in the caption for Figure 3, and the references. Nonetheless, we have duplicated this information in the text [L187-189]

Peak CO2 Levels by Room Functional Type and Building: *Context about rooms with wider CO2 level distributions might be valuable.* We respectfully note that this is beyond the scope of the manuscript. CO2 levels are highly complex, and depend on room volume, room occupancy, activity level, ventilation (both active and passive), seasonality, and a large variety of factors we intentionally did not measure. The focus of the manuscript was to estimate ventilation with real-world data, not to intricately model all the factors that might have contributed to CO2 levels.

Temperature versus CO2 Relationship: *Some interpretation or discussion regarding temperature fluctuations would enhance this section.* We have added to the existing discussion regarding temperature fluctuations [L214-215; L218-220; L222-223].

Air Changes per Hour Estimates: *Implications for ventilation could be discussed.* Thank you for your comment. This is most appropriately addressed in the Discussion section, rather than in Results, which we have done.

Incidence of SARSCoV2Positive PCR Tests: *Discussion of the practical implications is needed*. Thank you for your comment. This is most appropriately addressed in the Discussion section, rather than in Results, which we have done.

Overall Interpretation: *Should state the primary objective of the study more explicitly*. We have restated the primary objective [L268-270], which was also stated in the introduction.

Interpretation of Findings: *Needs to clarify the practical significance of not achieving an ACH ≥ 4. The caveats related to using CO2 to estimate ACH need to be concise.* We have addressed the recommendations of an ACH>4. Respectfully, while we done some editing for conciseness [L288-304], the issue of using CO2 to estimate ACH is complex, with a robust literature. Our discussion provides a number of critical caveats that would be important for a school measuring CO2 levels to consider for accurate use of this method, and to not overstate the utility of CO2 level measurements.

CO2 Monitoring: *Needs more explicit recommendations for using CO2 monitoring in schools. Suggests discussing the thresholds for CO2 levels. Could elaborate on the connection between students' ability to tolerate masking and CO2 levels.* Explicit recommendations for monitoring CO2 levels in schools are controversial, as noted by ASHRE. We have revised the manuscript to discuss this [L316-329]. Respectfully, we are not clear what "connection between the students ability to tolerate masking and CO2 levels" is referring to. Regardless of masking, expired CO2 will be the same, as masks are permeable to

CO_2; Masking is a neutral intervention in terms of CO_2 levels. Thus, we have not addressed this in the discussion.

Ventilation and Air Filtration: *Could be more concise and structured. The part about energy costs and outdoor air could be more concise.* Respectfully, all the issues briefly raised are critical considerations in terms of tradeoffs. In particular, there is only single sentence about outdoor air heating and energy costs, which is already concise [L336-338]. We have left this as is.

Community Engagement and Policy Implications: *Might benefit from specific examples or proposals. Consider elaborating on how the research partnership influenced the school.* Respectfully, specific examples at the end of the study would greatly expand the scope of the paper, without hard data. Rather than adding anecdotal examples, this will be the subject of an additional manuscript.

Conclusions: *A summary of key findings and implications is suggested.* We have added this.

Reviewer #3: *Authors investigated the correlation of classroom ventilation and exposure to CO2 with the incidence of SARS-CoV-2 over a 20-month period in a specialized school for students with intellectual and developmental disabilities. Study has deeply examined the strategies to keep the vulnerable populations of students in school while mitigating the risk of exposure and in-school spread of SARS-CoV-2 among students and staff. I appreciate the authors for studying such novel aspects in current scenario. Methodology, results and the discussion of the results are impressive. However, there are some minor corrections*

I suggest to improve the manuscript technically and scientifically sound. Highlight the research questions and key objectives (point-wise) at the end of the introduction part. We have revised the manuscript accordingly [L12-14].

Bring some more significant outcomes of the research that creates interest among the readers and include the future scope of the study in conclusion section. We have revised the discussion section to emphasize significant outcomes and to discuss the future scope of potential research.

Authors may also recommend or suggest some significant points to the policy makers/decision makers. We have revised the discussion section to cautiously suggest some points to policy makers. We are very mindful that as a single school study, in a very distinct population, it likely does not rise to the level that would warranted basing

Add some statistical bars (error bars or standard deviation) in the figures if possible. Respectfully, error bars were added when appropriate or relevant. Examples of CO_2 concentration measurements over time are not averages, and thus error bars are not appropriate [Fig 1, 3, 6]. Similarly, figures that contain histograms counts would not have error bars [Fig 2A, 7A]. Points denoting mean minimum and maximum ACH have standard deviations of <5% for all estimates, and error bars on each of 100 points would make the figures unreadable. Supplemental Figure 2 does show mean and standard deviation for each ACH estimate.
