# Supplementary material for: Ventilation during COVID-19 in a school for students with intellectual and developmental disabilities (IDD)
Source: PLoS One. 2024 Apr 3;19(4):e0291840. doi: 10.1371/journal.pone.0291840 (PMC10990219; doi:10.1371/journal.pone.0291840)
Supplement: Attachment — Submitted filename: Response to Reviewers Rev2.pdf [file pone.0291840.s005.pdf]

Response to Reviewers: PONE-D-23-26827: Ventilation during COVID-19 in a school for students with intellectual and developmental disabilities (IDD)

Response to Reviewers 1 and 3: We thank the reviewers for having recommended accepting the manuscript with no further revisions.

Response to Reviewer #2:

The article lacks chapter numbers throughout. Respectfully, the article was prepared according the PLoS One Instructions for Authors style guide, which does not require chapter numbers. Indeed, the sample manuscript body in the Instructions to Authors and the available templates provided on the web site, do not include this formatting. We have thus not changed the manuscript so that we remain within the format required by PLoS.

The figures lack titles and numbering, and the data within them contains three-line indications without corresponding labels for each line.

The Figures are submitted per the instructions for authors: "Do not include author names, article title, or figure number/title/caption within figure files. That information will go into your figure caption in the manuscript". Numbering is present in both the figure legends and in the upper right corner of the pages containing the figure itself in the reviewer's copy. Thus, to remain within the format required by PLoS, we have not changed the figures to include numbers and titles.

Additionally, the graphs utilize multiple colors for different lines. Please differentiate the graph lines using distinct styles.

The reviewer did not specify which graph is referred to, and so we have responded for each graph:

Figure 1 – Each panel has more than one line uses a different style: A) there are two lines in the graph, one solid with coloring explained in the figure legend, and the other dashed B) there is one solid line (fitted curve), and multiple data points. *We have not changed the figure*.

Figure 2 – Neither panel A or B contain "different lines" – A) is a histogram and B) is a scatter plot without a line. *As the comment does not appear to apply to this figure, we have not changed it.*

Figure 3 – Each panel with more than one line uses a different style: A) there are two lines in the graph, one solid with coloring explained in the figure legend, and the other dashed B) There are no lines, each is a scatter plot. *We have not changed the figure.*

Figure 4 – This is a stack graph, with no data lines. *As the comment does not appear to apply to this figure, we have not changed it.*

Figure 5 – There are three "lines" denoting the bands corresponding to CO2 level boundaries, clearly explained in the legend. All are dashed, and a corresponding color-coded bar on the y-axis serves as a second guide for the reader to the CO2 level bands. Using a distinct style for each boundary line would detract from the figure clarity. *We have not changed the figure.*

Figure 6 – These are time vs. value dot plots of the measurements. Given the measurement density, to plot them as lines that would be distinguishable by pattern (e.g. dashed, dot-dash, solid) would require significant down-sampling the data for smoothing, detracting from the accuracy and rigorous presentation of the data. *We have not changed the figure.*

Figure 7 – A is a histogram, and C is a biplot with a single superimposed line. *We have changed panel C so that line style differs among the ACH levels, as requested.*

Figure 8 – Panel A is a box-plot and B is are biplots, Neither panel contains “different lines”.
As the comment does not appear to apply to this figure, we have not changed it.

Refer to the following transaction journal and include it in the reference section that is more suitable for this article
1. Covid-19: automatic detection of the novel coronavirus disease from CT images using an optimized convolutional neural network.
2. The role of internet of things to control the outbreak of COVID-19 pandemic

Respectfully, we are very confused by this comment, and cannot discern if the reviewer is asking for (1) a different format for the bibliography along the style of these two articles, or (2) to include the references listed. First, and respectfully, we have formatted the reference section per the PLoS One Instructions for Authors using the LaTex. Second, our article does not refer at any point to medical imaging, the use of neural networks, or the internet of things, and so these references do not seem relevant. Is it possible that these comments might refer to a different manuscript than ours? If not, we would very much appreciate a clearer elucidation of these concerns.
